# Commentary: Adapting and Operationalizing the RE-AIM Framework for Implementation Science in Environmental Health: Clean Fuel Cooking Programs in Low Resource Countries

**DOI:** 10.3389/fpubh.2020.00218

**Published:** 2020-06-09

**Authors:** Ana A. Baumann

**Affiliations:** Brown School of Social Work, Washington University in St. Louis, St. Louis, MO, United States

**Keywords:** adaptation, implementation, low-middle income countries, framework, RE-AIM (reach, effectiveness, adoption, implementation and maintenance)

The field of implementation science has seen an accumulation of theories, models and frameworks in the past years. However, few empirical studies are informed by them (1), and when informed, few clearly describe how they applied the frameworks in the study (2). The study by Quinn et al. (3) provides an exception to this rule and gives us an example of how to use the Reach, Effectiveness, Adoption, Implementation, and Maintenance (RE-AIM) (4) framework in their study of a consortium of 11 sites in low middle income countries (LMIC). Instead of focusing on one study at a time, a consortium can advance the field by having common metrics across different settings, providing an unique opportunity for theory testing [e.g., (5, 6)].

## Testing the Boundaries of Construct Definitions

Quinn et al.' study (3) developed a checklist and case studies to evaluate household energy interventions. The results showed that the constructs *effectiveness* and *adoption* needed more adaptation in their definitions compared to the other constructs of the framework. *Effectiveness*, defined as “the impact of an intervention on important outcomes” (7), was hard to gather in their context because health is considered a co-benefit of the programs, and therefore health outcomes and measures of air pollutions are not usually readily available. To address this challenge, the authors adapted the definition to capture “potential” health impact of the stove/fuel, relying on estimated data accrued from stove emissions. We need more empirical studies in different contexts to continue to refine the definition of effectiveness.

*Adoption*, a construct used to capture the proportion and representativeness of organizations willing to adopt a program (7), was challenging because usually clean fuel cooking programs do not involve an intermediary organization. To address this issue, the authors re-defined the construct to encompass factors at society level (e.g., description of supply chain) as well as household/community factors (e.g., household use of technology). The *adoption* construct in this case was also difficult because it should refer not only to the uptake of something (i.e., adding a stove) but also to the discontinuation of older stoves who are health-damaging. This discussion is timely as the field starts to understand the unique aspects of de-implementation and how to define them. Accordingly, Prusaczyk et al. (8) suggest expanding the adoption concept to include *de-adoption*, defined as the intention or initial decision to stop a practice.

The results from Quinn et al. (3) showed that, while *Reach* was the easier construct to gather data across sites, the definition of reach is challenging in the context of public health programs. As Quinn et al. (3) comment: it is difficult to evaluate “Reach” of an intervention that improves sidewalks. Gaglio et al. (9) recognize the challenges in the definition, which has also been adapted to refer to awareness of a program (10). Finally, *Maintenance* was hard to capture because the sites were at the beginning of implementation the program. It will be interesting to see how this consortia captures maintenance later on.

## Context and LMIC

When stakeholders were asked about their perceived ease and usefulness of employing RE-AIM on their project, they mentioned the challenges in capturing context using RE-AIM, particularly the political and social aspects of the studies. In fact, as May et al. (11) state: “context is a problem in implementation science.” Let me explain.

Quinn et al. (3) mention that a solution to capture contextual outcomes could be using the Practical, Robust Implementation, and Sustainability Model (PRISM) framework, which is an expansion of RE-AIM. In fact, PRISM's constructs of *External Environment, Intervention, Implementation* and *Sustainability, Infrastructure*, and the *Recipients* align well with the RE-AIM constructs (12) and could be a great fit for Quinn et al.'s project.

However, as is shown by Quinn et al.'s data (3), we need to be careful about our assumptions that frameworks and constructs developed in high income countries (HIC) would fit in LMIC without any adaptation. This is because often contextual factors, such as health system structures, resource availability, cultural, and political norms and values are different in HIC compared to LMIC (13). In fact, the issues with fitting definitions of the implementation science constructs in LMICs are not unique to RE-AIM. In a systematic review of papers and authors survey, Means et al. (14) also identified challenges with some of the Consolidated Framework for Implementation Research (CFIR) (15) constructs. For example, similar to Quinn et al., their stakeholders also asked for more system level constructs, as they had difficulties applying the construct *patient needs* with interventions that took place at district or national levels. The examples of Quinn et al. and of Means et al. highlight the necessity of being humble with our frameworks, and to examine carefully our definitions to avoid the ethnocentric bias of implementation studies (16).

Several of us have written about the challenges of working in LMIC including issues with: (a) defining the evidence of the intervention (e.g., the fact that one intervention is proven efficacious in HIC, does not mean that it is efficacious in a LMIC), (b) measurement (i.e., issues of validity, availability of data), and (c) mechanisms of action (which may differ depending on context) (17, 18). As we continue to define our implementation constructs and outcomes, and better understand the theories and conceptual approaches, we should incorporate the testing of the boundaries of our implementation science frameworks in LMICs, as the majority of the frameworks and measures were developed in HICs. Perhaps now it is time for us to consider *how is implementation being conceptualized* (19). That is, in addition to adapting the definitions of the constructs of our frameworks, we should also have an explicit conversation about what is context and how context defines the boundaries of these definitions, our evidence, and who judges the usefulness of the frameworks and theories in which context. I look forward to more empirical studies so that we can continue to “theorize” (2) and contribute to the advancement of the field of implementation science.

## Author Contributions

The author confirms being the sole contributor of this work and has approved it for publication.

## Conflict of Interest

The author declares that the research was conducted in the absence of any commercial or financial relationships that could be construed as a potential conflict of interest.
